# Microbiological Evaluation of Local and Imported Raw Beef Meat at Retail Sites in Oman with Emphasis on Spoilage and Pathogenic Psychrotrophic Bacteria

**DOI:** 10.3390/microorganisms12122545

**Published:** 2024-12-11

**Authors:** Musallam A. Al-Mazrouei, Zahra S. Al-Kharousi, Jamila M. Al-Kharousi, Hajer M. Al-Barashdi

**Affiliations:** 1 Department of Food Science & Nutrition, College of Agricultural and Marine Sciences, Sultan Qaboos University, P.O. Box 34, Al-Khod, Muscat 123, Oman; s123572@student.squ.edu.om; 2 Applied Biotechnology Department, University of Technology and Applied Sciences, P.O. Box 411, Sur 411, Oman; 2019496022@utas.edu.om (J.M.A.-K.); 2019496016@utas.edu.om (H.M.A.-B.)

**Keywords:** aerobic plate count, cluster analysis, *E. coli*, coliform, hygiene, meat, psychrotrophic bacteria, VITEK, food safety

## Abstract

Determining the microbial quality and safety of meat is crucial because of its high potential to harbor pathogens. To address the critical knowledge gap and shed light on potential contamination risk in the meat supply chain, this study aimed to assess the underexplored microbial quality and safety of marketed beef meat in Oman. Thirty-three beef meat samples from six hypermarkets were analyzed for Aerobic Plate Count (APC), Psychrotrophic Bacteria Count (PBC), and coliform and *Escherichia coli* counts. Prevalences were 93% and 94% (means: 2.8 ± 1.1 and 2.6 ± 0.8 log CFU/g, respectively) for coliform, and 80% and 83% (means: 1.8 ± 1.4 and 1.7 ± 0.9 log CFU/g, respectively) for *E. coli* in imported and local samples, respectively. The mean counts of APC (6.3 ± 0.1 log CFU/g) and PBC (6.2 ± 0.2 log CFU/g) were statistically similar but different from those of coliform and *E. coli*. Bacterial identification using VITEK 2 compact revealed spoilage bacteria (*Pseudomonas luteola*, *Pseudomonas fluorescens*, and *Shewanella putrefaciens*) and pathogenic bacteria (*Acinetobacter bumannii* complex, *Aerococcus viridans*, *Enterococcus faecalis*, and *Oligella ureolytica*), which demonstrates a potential for both spoilage and pathogen-related risks. It is concluded that the APC counts of all samples exceeded acceptable standards set by the G.C.C. Standardization Organization (GSO), which was established to protect food safety and public health in Oman and other Gulf countries. This suggests an increased risk of spoilage and pathogen contamination. This study provides one of the earliest reports of microbial contamination levels in meat, serving as an eye-opener for policymakers and stakeholders. It highlights a need for stricter hygiene protocols and improved meat handling and processing practices to enhance meat safety and protect public health in Oman and the Gulf region.

## 1. Introduction

Although meat is one of the most important nutrient-dense and energy-packed natural foods [1], data on its microbial quality and safety are largely lacking in Oman. According to the National Center for Statistics and Information (NCSI), consumption of red meat is increasing in Oman, which reached a total of 400 million Kg in 2021 compared to 60 million Kg in 2000 [2]. Bacteria can contaminate meat and meat products at any time from production to consumption [3] and proliferate in meat’s fertile environment, making it a high-risk food. These bacteria can be pathogens, opportunistic pathogens, or commensals that can also be reservoirs for antibiotic-resistant genes or other virulence genes. Animal-source foods are the leading cause of foodborne diseases, and they are expected to continue harboring pathogens that will cause outbreaks and deaths in the future because no effective interventions can eliminate them from food animals or other foods [3].

If meat is not stored and prepared properly, pathogenic bacteria can multiply and cause infections. Although meat is usually cooked, some people eat meat dishes that are rarely cooked such as meat steaks where the heat does not reach the center of the meat. Moreover, bacteria can be transferred through cross-contamination from meat to other foods that are eaten raw such as fresh fruits and vegetables [4]. Bacteria may produce heat-resistant toxins that can withstand cooking temperatures or produce spores that can survive high temperatures and germinate later [5,6]. Various pathogenic and indicator bacteria were reported in meats in different countries. For instance, in Ethiopia, 120 samples were collected from three local markets from August 2020 to March 2021. *Escherichia coli* (16.67%) was dominant, and the coliform count was 3.95 log CFU/g. Researchers indicated that the microbiological quality and safety of meat were poor and recommended the regular monitoring of meat and training of all personnel involved in food handling [7]. Similarly, another study in Pakistan determined the microbiological quality of 30 raw meat samples from beef, mutton, and poultry collected from different shops of butchers. Coliforms were detected in 80% of mutton and beef samples, suggesting poor environmental sanitation and unhygienic meat handling [8]. Aerobic Plate Count (APC), which is a critical indicator of the hygienic quality of a product throughout processing and distribution [9,10,11], was found to be 7.15, 6.92, and 6.62 log CFU/cm^2^, respectively, for the meat of beef, sheep, and goats at the retail outlets in Lahore, while the *E. coli* count in beef, sheep, and goats were 2.64, 2.78, and 2.86 log CFU/cm^2^, respectively. These counts were considered high enough to pose risks of spoilage and food-borne illness [12]. Studies in Iraq on frozen meat reported APCs in some markets as high as 5.4 and 6.1 log CFU/cm^2^. These counts were considered unacceptable with emphasis on the importance of the proper transportation and storage of meat [13]. These studies collectively highlight widespread issues of microbial contamination in meat and underscore the need for better hygiene practices and regular monitoring in the mentioned places and probably in the neighboring geographical areas.

Coliforms belong to gut bacteria and their count is used as an index for hygiene [14,15]. Pathogenic *E. coli* is an important Gram-negative foodborne pathogen that has global health concerns. *E. coli* is also a normal inhabitant of the intestinal tract of humans and many animals and birds and is used as an index microorganism for the possible presence of enteric pathogens in water and food and as an index for fecal contamination [16]. Fresh raw meats are usually stored at low temperatures. Therefore, Psychrotrophic Bacteria Counts (PBCs) can be useful indicators for the hygienic condition of meats at low temperature and their spoilage potential. Their high counts may indicate a suitable environment for the growth of pathogens such as *Listeria monocytogenes,* which is a food-borne pathogen that causes listeriosis with high mortality rates. Its control in food production areas is challenged by its ability to grow in low-temperature, aerobic, anaerobic, and modified packaging systems [17].

The VITEK system is widely accepted to be utilized for the routine identification of clinical, environmental, and food microbes [18]. The identification of bacteria isolated from different selective and non-selective media is critical to confirm their identity and possible involvement in causing diseases for the public and to evaluate hygiene practices to establish appropriate monitoring and control measures. Cluster analysis effectively visualizes the relatedness of foodborne microbial isolates by grouping them based on similarities in their biochemical profiles. This approach highlights patterns in metabolic and functional traits among different food-associated microbes, allowing for a clearer understanding of their phenotypic relationships. By organizing these isolates into clusters, strains can easily be assessed in terms of their biochemical behavior in food environments, offering insights into their roles in food spoilage, safety, and even other beneficial roles such as fermentation and preservation.

The Middle East and North Africa are classified with the third highest number of food-borne disease cases per population [9]. While the microbial contamination of meat has been studied in various countries, information specific to the microbial quality of meat, the prevalence of *E. coli*, and coliform in food animals in Oman is limited. The current study aimed to address this knowledge gap by evaluating the microbial quality of local and imported beef meats marketed in Oman. In particular, the objectives were to utilize standard microbial methods of pour and spread plate techniques to perform APC, PBC, and *E. coli* and coliform counts in local and imported meats available at retail sites in Oman, to compare the microbial count of local and imported meats, to compare the microbial counts between different hypermarkets, and to identify psychrotrophic bacteria using their biochemical profile reported by the automated machine VITEK 2 Compact 15. This paper also describes a method for organizing and analyzing large datasets of biochemical results generated by VITEK for cluster analysis or dendrogram construction for better visualization and interpretation of the data. Findings from this study may help identify the role of raw marketed red meat in the dissemination of *E. coli*, coliforms, and emerging pathogens and give recommendations to enhance the hygienic standards for meat production in the examined sites to contribute to consumers’ health protection.

## 2. Materials and Methods

### 2.1. Sample Collection

The randomization approach for selecting samples aimed to minimize bias by purchasing samples without predetermined selection criteria from each hypermarket. Upon arrival at each location, samples were chosen based on availability, without preference for specific brands, cuts, or packaging types. This method ensured a representative sampling from each hypermarket, reflecting the diversity of imported and local raw beef available to consumers in Muscat during the collection period (February to April 2024). Thus, thirty-three raw beef samples (15 imported and 18 local) were randomly selected and purchased from six different hypermarkets in Muscat Governorate. To maintain the cold chain integrity, samples were transported with ice packs to maintain a low temperature during transit to the laboratory, which took about half an hour, where they were immediately analyzed. The selected hypermarkets were labeled from 1 to 6 and were chosen because they are among the most popular shopping destinations in Muscat, frequented by a large segment of the population. They represent key points of purchase for a majority of consumers in the area, providing a reliable cross-section of the raw beef products widely available to the public. Their popularity ensures that the samples reflect products accessible to a broad demographic across various socio-economic backgrounds.

### 2.2. Sample Preparation and Microbial Counts

Meat samples were analyzed in a safety cabinet (Purifier class II, Labconco, Kansas, MO, USA) and cut into small pieces using sterile scalpels. Twenty-five grams of the cut sample was weighed in a sterile stomacher bag, mixed with 225 mL of Maximum Recovery Diluent (MRD), and homogenized for 1 min using a stomacher (Bagmixer 100 MiniMix, Interscience, Bois Arpents, France). Serial dilutions were prepared from the original homogenate in MRD. Aerobic Plate Count (APC) and Psychrotrophic Bacteria Count (PBC) were performed by the spread plate method on Tryptone Soy Agar (TSA). Plates were incubated at 35 °C for 72 h for APC [10] and at 10 °C for 7 days for PBC [11]. The count of coliform was performed on Violet Red Bile Lactose (VRBL) agar by the pour plate method [12]. Briefly, one milliliter of each dilution was transferred to a Petri dish separately by a sterile pipette, and then the medium was poured after cooling to a temperature of 45 °C and the Petri dishes were rotated to distribute bacteria evenly and left to solidify. The plates were inverted and incubated at 35 °C for 24 h. Tryptone Bile X-glucuronide (TBX) medium was used to count *E. coli* [10]. The plates were incubated at 35 °C for 24 h. *E. coli* forms blue/green colonies on TBX while typical coliform colonies appear red/purple on VRBL. More details on the morphology of typical bacterial colonies on TBX and VRBL can be searched on the manufacturer’s website [19]. All microbiological media were from Oxoid, Basingstoke, UK.

### 2.3. Identification of Bacteria and Biochemical Analysis

Depending on their morphology, one to three colonies were selected from each sample for identification. The confirmation of *E. coli* obtained on TBX and psychrotrophs was carried out for some colonies after purification on TSA and incubation at 35 °C for 24 h for *E. coli* and 24–48 h for psychrotrophs. Confirmation was carried out to check the selectivity of the medium incubated in the described conditions. Bacteria were identified biochemically using automated identification equipment, VITEK 2-compact 15 (bioMérieux, Marcy-l’Étoile, France), according to the manufacturer’s method. Briefly, bacterial suspensions were prepared in sterile saline (0.45%) and the density was adjusted to a McFarland (McF) standard of 0.5–0.63 using VITEK 2 DensiCheck (bioMérieux, France). The DensiCheck device was calibrated with the McF standard of 0, 0.5, 2, and 3 glass tubes provided by bioMérieux (France). *E. coli* ATCC 25922 and *Staphylococcus aureus* ATCC 25923 were used as control strains to validate the accuracy of bacterial identification using VITEK 2-compact 15. The GN and GP cards were used after confirming that the isolated bacteria are Gram-negative or Gram-positive, respectively, using the Gram stain [10]. The types of biochemical tests included in 47 wells of GN cards and 43 wells of GP cards are displayed in Table 1 and Table 2, respectively.

### 2.4. Data Analysis

Statistical analysis of the data was carried out using the SAS statistical software package (JMP^®^ SAS 17.2.0, 2022–2023, Cary, NC, USA). One-way analysis of variance (ANOVA) was used to determine whether there were significant differences between different counts (APC, PBC, coliform, and *E. coli*) in general and according to the origin of meat samples (local or imported) and the hypermarket. Differences were considered significant if *p* < 0.05. The Tukey–Kramer HSD test was used to detect the source of differences when they were detected by ANOVA. In addition, the results of the biochemical tests for the Gram-negative bacteria isolated in the current study, a reference strain (*E. coli* ATCC 25922), and an *E. coli* strain previously isolated from pistachio (unpublished data) were analyzed for similarity by constructing a dendrogram through hierarchical clustering (method: Ward linkage) [20] using the mentioned SAS statistical software package. The analysis included 423 results (310 negative and 113 positive results) for 47 biochemical tests (Table 1). A method is shown in Figure S1 for transferring raw data from PDF files generated by VITEK to Excel files for further analysis. In addition, Generative Artificial Intelligence (AI) programs such as ChatGPT can be used for this purpose.

## 3. Results

### 3.1. Microbial Counts

**Figure 1:**
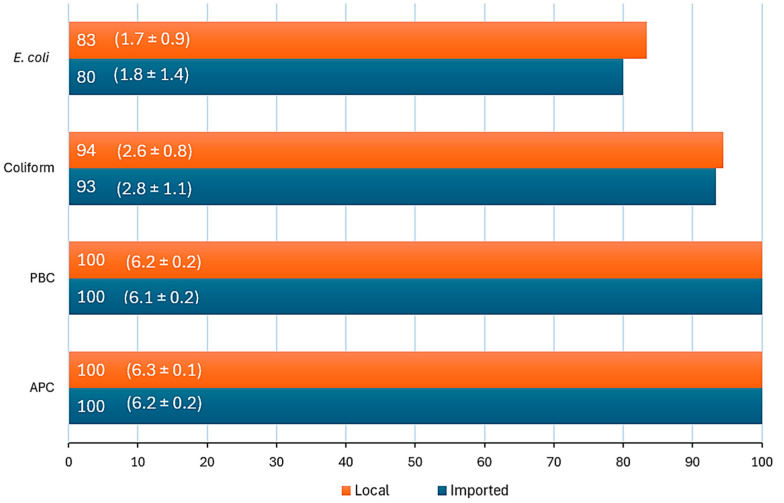
Percentage of positive samples for Aerobic Plate Count (APC), Psychrotrophic Bacteria Count (PBC), coliform, and *E. coli* counts in local and imported raw beef meat samples. Means of counts ± standard deviations are shown in parentheses (log CFU/g).

It was noted from the general observations of meat handling during sample collection that some samples had unpleasant odors and that the adherence to the hygiene practices such as cleaning cutting boards and knives after each use and wearing aprons and hairnets varied among hypermarkets. In this study, APC was determined because it indicates the total microbial load in meat in which high APC levels may indicate poor hygiene during processing, storage issues, or contamination risks [10,11]. PBC measures bacteria capable of growing at refrigeration temperatures, which is critical for assessing spoilage potential. Elevated PBC levels may signify improper cold storage or extended storage durations [6]. Coliforms are used as indicator organisms for fecal contamination and hygiene. High coliform counts suggest unsanitary handling practices or contamination during slaughter and processing [15]. *E. coli* is a specific indicator of fecal contamination and poor hygiene practices [16]. Figure 1 shows the percentages of positive samples for APC, PBC, coliform, and *E. coli* counts, highlighting the microbiological quality of both local and imported samples. Notably, APC and PBC were positive for all local (*n* = 18 samples) and imported (*n* = 15) samples, reflecting the widespread presence of aerobic and psychrotrophic bacteria. Coliform detection was high in both local (94%) and imported (93%) samples, with mean values (2.8 ± 1.1 and 2.6 ± 0.8 log CFU/g, respectively) suggesting moderate contamination levels. Similarly, the prevalence of *E. coli* was slightly higher in local samples (83%) compared to imported ones (80%), with comparable mean counts (1.7 ± 0.9 vs.1.8 ± 1.4 log CFU/g, respectively). This similarity suggests that both sources might face similar contamination risks during processing or transport. These findings underline the need for improved food safety measures across all sample sources.

**Figure 2:**
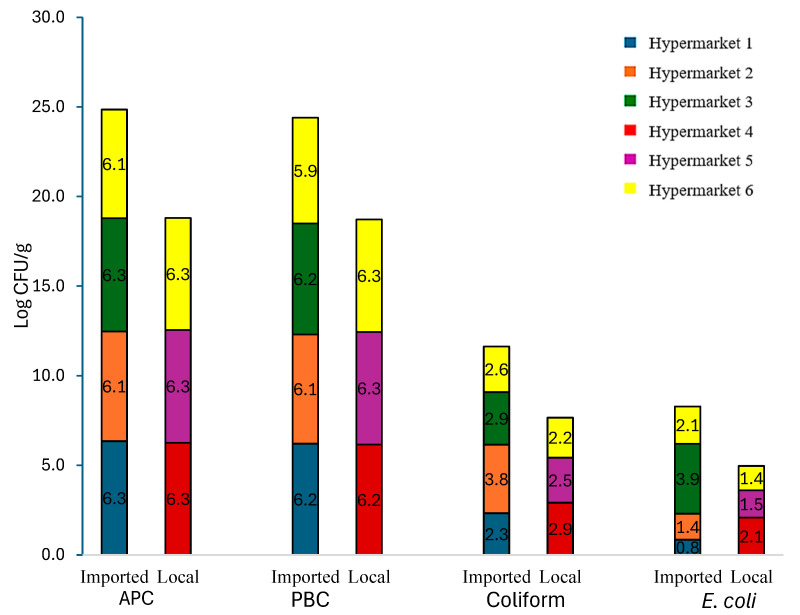
Averages (Log CFU/g) of Aerobic Plate Count (APC), Psychrotrophic Bacteria Count (PBC), *E. coli*, and coliform counts in imported (4 markets: 1, 2, 3, and 6) and local (3 markets: 4, 5, and 6) meat samples.

Figure 2 presents the microbial counts (APC, PBC, coliform, and *E. coli*) across different hypermarkets for local and imported meat samples. The APC and PBC had the highest counts, followed by coliforms and *E. coli*, reflecting the general microbial load and distribution in the samples. ANOVA indicated significant differences between the microbial counts (*p* < 0.0001), with the Tukey–Kramer HSD test grouping APC (6.3 ± 0.1 log CFU/g) and PBC (6.2 ± 0.2 log CFU/g) together, coliform (2.7 ± 1.0 log CFU/g) separately, and *E. coli* (1.7 ± 1.1 log CFU/g) in a third group.

Microbial counts (APC, PBC, coliform, and *E. coli*) did not differ significantly between local and imported meat samples (*p* = 0.5610, 0.0634, 0.4889, and 0.6738, respectively), indicating comparable microbial quality in both categories. However, *E. coli* counts varied significantly among hypermarkets (*p* = 0.0083). The Tukey–Kramer HSD test showed that markets 1, 2, 5, and 6 had lower *E. coli* counts, market 3 had higher counts, and market 4 had intermediate counts overlapping with both groups. These differences suggest variability in hygiene or handling practices between hypermarkets. No significant differences were observed in APC, PBC, or coliform counts across hypermarkets (*p* = 0.1187, 0.7716, and 0.2274, respectively), indicating consistent levels of these indicators in all markets. These findings highlight the need for improved hygiene to minimize microbial contamination and ensure consistent safety standards across hypermarkets, thereby protecting consumer health.

### 3.2. Bacterial Identification

Among Gram-negative bacteria, three isolates of psychrotrophic bacteria obtained from different samples (two imported and one local) were identified as *Shewanella putrefaciens* (Table 3) with different bionumbers. Two isolates (one from a local sample and the other from an imported sample) were identified as *Pseudomonas fluorescens* and one isolate was identified as *Pseudomonas luteola*. *Acinetobacter baumannii complex* (*A. nosocomialis*, *A. pittii*, *A. baumannii*, and *A. calcoaceticus*) and *Oligella ureolytica* were isolated from local samples. Two isolates (one from a local sample and the other from an imported sample) grown on TBX medium were confirmed as *E. coli* with different bionumbers. Among Gram-positive bacteria, *Enterococcus faecalis* was identified in an imported sample and *Aerococcus viridans* in a local sample (Table 3). These results highlight the diversity of bacterial species present in both local and imported meat samples, including potential spoilage organisms and pathogens. The identification of psychrotrophic bacteria and *E. coli* underscores the importance of stringent handling and storage practices to ensure meat safety and minimize health risks to consumers.

### 3.3. Cluster Analysis

**Figure 3:**
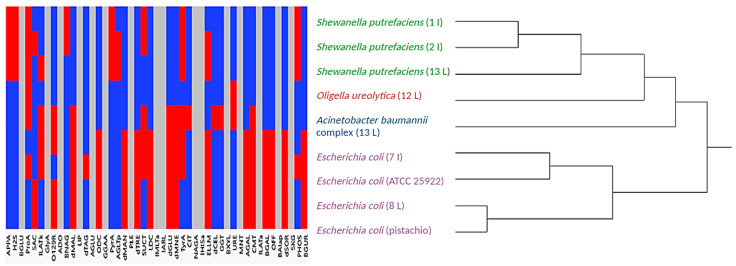
Dendrogram showing a hierarchical clustering of bacteria identified in this study, the reference strain *E. coli* ATCC 25922, and *E. coli* isolated from a plant source (pistachio), according to their biochemical profile determined by VITEK^®^ 2 GN (very good identification). 1, 2, 7, 8, 12, and 13 are the sample numbers. L: local, I: imported samples. Abbreviations of the 47 biochemical tests are represented in Table 1. Blue indicates positive results of a particular test for some isolates, red indicates negative results of a particular test for some isolates, and gray indicates negative results of a particular test for all isolates.

Cluster analysis of the biochemical profiles revealed interesting relationships among isolates from different sources. For instance, *E. coli* isolated from the local meat sample 8 shared the same bionumber as *E. coli* from an imported pistachio sample (Table 2) and clustered together in the dendrogram (Figure 3). This suggests potential cross-contamination or a shared contamination source, such as handling equipment, water, or environmental conditions in the processing or distribution chain. Similarly, *S. putrefaciens* isolates from imported samples 1 and 2 formed a cluster with *S. putrefaciens* from the local sample 13, indicating that this bacterium might persist across diverse food sources, potentially due to similar environmental niches or processing conditions. The clustering of *E. coli* from imported sample 7 with the reference strain (*E. coli* ATCC 25922) in a sub-cluster, closely related to the sub-cluster of *E. coli* from local sample 8 and pistachio, further supports the hypothesis of shared contamination pathways or similar selective pressures acting on these strains. The separation of *A. baumannii* complex and *O. ureolytica* from all other clusters suggests distinct biochemical characteristics and likely unrelated contamination sources. Moreover, the dendrogram’s color-coded representation of biochemical test results highlighted that all bacteria shared negative results for 16 tests, suggesting common metabolic limitations, while variations in the remaining tests (indicated by blue and red colors) point to differences in their ecological adaptability.

These findings underscore the importance of cluster analysis in identifying contamination patterns and potential links between seemingly unrelated food sources. The similarities in biochemical profiles suggest that cross-contamination might occur at shared points in the supply chain or through common reservoirs, emphasizing the need for stringent hygiene and monitoring practices to minimize risks.

## 4. Discussion

The microbial quality and safety of meat in any country need continuous and regular monitoring and updates, which is also important for industry to ensure consumers are provided with high-quality and safe meat and meat products [1]. This study evaluated the microbial quality of local and imported beef samples sold in six hypermarkets by assessing APC, PBC, coliform, and *E. coli* counts. All samples exhibited high APC levels (means: 6.2 ± 0.2 and 6.3 ± 0.1 CFU/g for imported and local samples, respectively). Coliforms were detected in 93% and 94% of imported and local samples, with mean counts of 2.8 ± 1.1 and 2.6 ± 0.8 CFU/g, respectively. Psychrotrophic bacteria were prevalent in all samples (means: 6.1 ± 0.2 and 6.2 ± 0.2 CFU/g for local and imported samples). The prevalence of *E. coli* was 80% in imported and 83% in local samples, with counts significantly impacted by the hypermarket of origin. This study also identified spoilage bacteria (*S. putrefaciens*, *P. fluorescens*, and *P. luteola*) and pathogens (*A. baumannii* complex, *O. ureolyticus*, *A. viridans*, and *E. faecalis*). While spoilage bacteria contribute to the deterioration in meat quality, the presence of pathogens poses a direct risk to consumer health. These pathogens can cause foodborne illnesses, emphasizing the critical need for improved hygiene and monitoring practices in meat production to mitigate both spoilage and health risks. APC indicates the hygienic quality of a product throughout processing and distribution [10,11] and is often used to evaluate the overall microbial quality and shelf life of meat [6,21]. Some researchers [22] reported a strong association between APC and the presence of *E. coli* in beef carcasses where 88% of samples with an APC of ≥4 log CFU/cm^2^ were positive for *E. coli* while only 21% of samples with an APC of <2 log CFU/cm^2^ were positive. In this study, all samples had an APC of ≥6.0 CFU/g and *E. coli* was detected in most samples (Figure 1). Coliforms were even more prevalent than *E. coli* and were detected in the majority of samples (≥93% of samples). This is expected as various bacterial genera such as *Citrobacter*, *Enterobacter*, *Klebsiella*, and *Serratia* are included in the group of coliforms [14]. 

All samples in this study supported the growth of psychrotrophic bacteria at 10 °C. Unlike psychrophilic microbes, which thrive in permanently cold environments with a maximum growth temperature of 20 °C or below, psychrotrophic bacteria can grow at temperatures exceeding 20 °C and are widely found in foods and natural habitats [23,24]. These bacteria, capable of growing at 7 °C or lower, often exhibit optimal growth below 30 °C [6]. Psychrotrophic bacteria possess adaptations, such as functional proteins at low temperatures and elevated levels of unsaturated fatty acids in their cell membranes, enabling them to grow in cold environments. While their activity in foods may result in spoilage or pose a risk of infection, they also play a crucial role in biodegradation in natural ecosystems, particularly during colder seasons [23].

The APC and PBC in the studied samples were statistically similar, reflecting the ability of meat-borne microbes to grow across a range of temperatures. This similarity suggests that the meat samples may not have been stored at refrigeration temperatures for extended periods. Freshly slaughtered meat typically supports mesophilic bacterial growth, whereas prolonged cold storage fosters psychrotrophic growth. Comparable findings were reported in broiler carcasses where APC decreased during cold storage, but PBC did not [25,26]. In this study, all APCs exceeded the acceptable limit of ≥6.0 log CFU/g as per the G.C.C. Standardization Organization (GSO) [27] and the International Commission on Microbiological Specification [28]. These high counts align with previous studies on fresh and frozen meat, such as research from Iraq and Egypt, which reported APC values exceeding the acceptable standards for raw meat [13,28]. The APC reflects the overall microbial load in the samples, which, when exceeding acceptable limits, indicates significant microbial activity. This microbial activity can result in the degradation of meat quality through the consumption of nutrients, leading to the production of undesirable volatile compounds. Such spoilage renders the meat unsuitable for consumption and could also result in economic losses for retailers and consumers.

Meat’s perishable nature stems from its high nutrient content and water activity. While low-temperature storage delays spoilage by slowing microbial activity, psychrotrophic bacteria such as *Pseudomonas*, *Yersinia*, and *Listeria* can contribute to spoilage or cause infections [6,29]. The PBC, which represents psychrotrophic bacteria capable of growth at refrigeration temperatures, is particularly significant in cold-stored foods. These bacteria, including species like *Pseudomonas*, can dominate spoilage processes, producing off-odors, slime, and discoloration in meat. Their ability to thrive under refrigeration conditions underscores the importance of maintaining strict hygiene during meat processing and storage to limit their growth. High PBCs in this study may indicate inadequate hygiene during slaughtering, processing, or retail handling.

While coliform levels differed significantly from APC, PBC, and *E. coli* counts, the highest coliform count (3.8 log CFU/g) was observed in imported meat, consistent with prior findings in Afghanistan [30]. While coliform bacteria are not the primary drivers of spoilage, their presence indicates inadequate hygiene or potential cross-contamination during processing or storage. Lower coliform counts compared to APC and PBC could be attributed to the nature of coliforms, which may not thrive as effectively under cold storage conditions as psychrotrophic bacteria. Additionally, while the APC encompasses a broad range of microbes, coliforms are a specific subset that represents a smaller proportion of the overall microbial community in meat.

Pathogenic *E. coli*, a key foodborne pathogen, serves as an indicator of fecal contamination and the potential presence of enteric pathogens [16,31,32]. Elevated *E. coli* counts in meat are a direct food safety concern. The detection of *E. coli* may signal lapses in sanitary practices during slaughter, processing, or retail handling, as these bacteria originate from the intestinal tract of animals. Foods may also harbor extraintestinal pathogenic *E. coli* (ExPEC), associated with hospital- and community-acquired infections. ExPEC’s ability to colonize the intestine for extended periods and cause infections under specific conditions complicates tracing its sources. Evidence suggests a link between human ExPEC and avian pathogenic *E. coli*, implicating foods as reservoirs [16]. The prevalence of antibiotic resistance in meat-associated *E. coli* is a concern, as exemplified by findings from Ghana, where 86.67% *of E. coli* isolates from beef exhibited resistance [33].

In this study, hypermarket practices significantly influenced *E. coli* counts but not APC, PBC, or coliform counts. Lower *E. coli* counts were observed in hypermarkets 1, 2, 5, and 6, while hypermarket 3 showed the highest counts, potentially reflecting variations in hygiene practices [34]. Imported beef samples demonstrated higher *E. coli* contamination compared to local samples, consistent with a previous study in Iraq that revealed unacceptable *E. coli* levels in imported samples [35]. Researchers concluded that local meat is preferable to imported meat and recommended stricter safety regulations for imported meat. Researchers attributed higher *E. coli* levels in imported samples to extended transit times and improper handling or storage during importation. Prolonged transportation might increase the likelihood of contamination due to temperature fluctuations, cross-contamination, or inadequate hygiene during handling in multiple stages of the supply chain. Further investigation would be necessary to identify specific factors contributing to this finding. However, no significant differences were observed in APC, PBC, or coliform counts between imported and local samples, suggesting similar hygienic conditions across these meat sources. Similarly, no significant differences in APC counts were reported between locally produced and imported fresh fruits and vegetables in Oman [10], indicating a similar contamination level with total aerobic bacteria in both animal and plant origin foods.

Meat is spoilt by what is known as specific spoilage organisms (SSOs), which include bacteria such as *Pseudomonas* spp., lactic acid bacteria, *Enterobacteriaceae*, *Acinetobacter* spp., *Aeromonas* spp., *Moraxella* spp., *Micrococcus* spp., and many others that can produce metabolites that change the sensory properties of meat and negatively affect its quality making it unfit for human consumption [36]. In this study, VITEK revealed the identification of three isolates of psychrotrophic bacteria obtained from different samples (two imported and one local) as *S. putrefaciens*. Two isolates (one from a local sample and the other from an imported sample) were identified as *P. fluorescens* and one isolate was identified as *P. luteola* (Table 1). *S. putrefaciens* is known for its ability to spoil meat and other protein-rich foods. Also, it is known for producing trimethylamine (TMA), which is responsible for the fishy odor in spoiled meat [37]. *S. putrefaciens* can grow at low temperatures, including refrigeration temperatures (4–10 °C). While its growth rate is slower at temperatures below 10 °C, it can still proliferate and cause spoilage over time [38]. *Pseudomonas* species is considered one of the principal bacteria that causes meat spoilage because it produces fat and protein hydrolases and biosurfactants [29]. *P. fluorescens* and *P. luteola* have been frequently isolated from meat and meat products. They are known for their ability to spoil raw meat and other protein-rich foods and produce off-odors in spoiled meat. These odors are primarily due to the production of volatile compounds like ammonia and hydrogen sulfide. In addition, these bacteria can produce extracellular polysaccharides, leading to slime formation on the meat surface, which is a common indicator of spoilage that occurs under aerobic conditions and leads to the rapid spoilage of meat exposed to air. The biofilms also make these bacteria difficult to eradicate by standard cleaning agents and sanitizers [36].

*A. baumannii* complex (*A. nosocomialis*, *A. pittii*, *A. baumannii*, *A. calcoaceticus*) was isolated from a local sample. After being low-grade pathogens, *A. baumanii* complex (*A. nosocomialis*, *A. pittii*, *A. baumannii*, and *A. calcoaceticuis*) has recently become of critical importance as bacteria in this complex have appeared as emerging pathogens causing severe nosocomial infections such as pneumonia with significant rates of mortality [39]. This is because of its potential to resist antibiotics and adapt to various environments. Information about the presence of *A. baumanii* complex bacteria outside hospitals is still limited although there is an increasing number of reports of its presence in new habitats such as soil, water, domestic and wild animals, vegetables, and food animals [40]. In China, 22 strains of *A. baumannii* were isolated from 126 samples of meat intended for human consumption. In another study conducted in Saudi Arabia [41], 55 strains of *A. baumannii* were isolated from 220 samples of various types of meats including camels, chickens, cows, and sheep. The isolation of bacteria within this complex in this study from raw meat sold for consumers adds to this list and isolating them from psychrotrophic plates highlights the capability of this pathogen to proliferate at cold temperatures. Thus, monitoring the presence of unusual food-contaminating bacteria is critical to understanding any new trends in the spread of pathogens through the food chain. This might require using nonselective media, enrichment techniques, or an analysis of the sample microbiome and not only targeting specific bacterial pathogens.

This research reports the presence of the pathogen *O. ureolytica* in meat. This bacterium belongs to the *Alcaligenaceae* family and it can cause respiratory and urinary tract infections and wound infections. Recently, it was described as causing a lethal infection in an elderly woman in France where the treatment with multiple antibiotics failed [42]. The meat source of this bacterium in this study is locally produced. Meat can be one of the sources or vehicles for *O. ureolytica*. In the literature, this pathogen is more related to opportunistic nosocomial infections. It would be interesting to study its prevalence as a foodborne bacterium and investigate its genetic makeup and pathogenicity.

The Gram-positive bacteria *A. viridans* and *E. faecalis* were identified in local and imported raw meat samples, respectively. *A. viridans* is an important zoonotic pathogen with increasing antibiotic resistance in recent years. It threatens animals and causes various diseases such as mastitis in dairy cows [43], but it also causes infections such as arthritis, endocarditis, bacteremia, and meningitis in humans. Some investigators [44] reported the first identification (using Matrix-Assisted Laser Desorption/Ionization–Time-of-Flight Mass Spectrometry; MALDI-TOF MS biotyper) of *A. viridans* from goat meat that was found to contain residues of antibiotics and noted that the presence of antibiotics in meat might select this pathogen as it possesses resistance to various antibiotics. In a study conducted in Turkey, 93 isolates of enterococci were identified in 100 ground beef samples of which 72.04% were *E. faecalis* and their genetic characterization revealed the presence of 41 pulsed-field gel electrophoresis (PFGE) patterns that were classified into 15 clusters [45]. In Switzerland, 17 isolates of *E. faecalis* were isolated from 24 samples of commercial-raw-meat-based diets intended for companion animals. The presence of this pathogen in meat offered raw to pets with the presence of various antibiotic resistance genes poses a risk to the pets and their owners [46].

This research describes a method that can be used to analyze hundreds or thousands of biochemical results produced by the automated machine VITEK 2 compact simultaneously. This enables better visualization and presentation of the results for comparison between different isolates and investigating their relatedness based on their biochemical profiles. It is recommended to use the Ward linkage method because it joins the two clusters that produce the minimum variance in the joined cluster, thus producing compact clusters by minimizing the sum of squares within clusters which is advantageous when analyzing biochemical profiles. Although the determination of genetic relatedness is crucial, using this technique besides genetic methods will provide valuable data about the functionality of the genes encoding them. Although previous research found MALDI-TOF MS (analysis is based on bacterial protein spectra) to be a more accurate method for enterococci species identification than VITEK 2 (analysis is based on biochemical patterns), the dendrograms obtained from the two systems placed enterococci isolates in identical positions [47]. In the current study, cluster analysis placed *E. coli* isolated from local meat sample 8, which had the same bionumber as *E. coli* isolated from imported pistachio (Table 3), in the same cluster (Figure 3) with the shortest distance in the dendrogram, which reflects the number of matching positive and negative results of the biochemical results. The two isolates of *S. putrefaciens* isolated from different samples (imported samples 1 and 2) clustered together with the second shortest distance between two isolates in the dendrogram and close to *S. putrefaciens* isolated from the local sample 13. *E. coli* isolated from imported sample 7 clustered with *E. coli* ATCC 25922 with the longest distance between any two isolates clustered together in the dendrogram. As expected, *A. baumannii complex* and *O. ureolytica* separated from other clusters (Figure 3). The color map of the results of the biochemical tests can be quickly invaginated to check any patterns; for example, all isolates had negative results for 16 biochemical tests (gray bars) or to check the results of specific tests such as susceptibility to O129 (Figure 3).

The findings in this study highlight significant food safety concerns. The high prevalence of *E. coli*, coliforms, and psychrotrophic bacteria indicates inadequate hygienic practices during slaughter, processing, and storage. The identification of spoilage organisms emphasizes the importance of strict cold chain management to delay spoilage and maintain meat quality. Emerging pathogens like *A. baumannii* complex and *O. ureolytica* in meat products call for enhanced monitoring protocols, including microbiome analyses, to detect unusual contaminants. This study demonstrates the utility of automated identification methods like VITEK 2 for biochemical profiling. While MALDI-TOF MS has been shown to be more accurate for certain pathogens, combining these techniques with genetic methods can provide comprehensive insights into both genetic and functional characteristics. This study was limited to six hypermarkets in one region, which may not represent the broader meat supply chain. Additionally, genetic analyses were not performed to confirm the relatedness of isolates except those collected and identified in the preliminary experiments through the sequencing of the 16S rRNA gene of five bacterial isolates obtained from fresh meat samples sold in hypermarket 2 and grown on psychrotrophic plates as described in this study. Genetic analysis was carried out as previously described elsewhere for sequencing the 16S rRNA gene [48]. Genetic sequencing identified these isolates as *Pseudomonas fragi* (two isolates), *Pseudomonas psychrophila*, and *Brochothrix thermosphacta* in the local sample and *Carnobacterium maltaromaticum* in the imported sample. These findings reinforce the results obtained from phenotypic identification and highlight the presence of psychrotrophic spoilage bacteria in the samples [49,50]. The molecular data provide an additional layer of validation, underscoring the need for further integration of genotypic methods in microbial quality assessments. Accession numbers for the identified isolates from the preliminary study have been provided by GenBank (PQ620248-PQ620252), contributing to the broader understanding of spoilage-associated microbiota in meat products.

Future research should expand geographic coverage and incorporate genetic characterization to explore pathogen transmission dynamics. More samples, bacteria, and pathogenic bacteria can be targeted in the future. Enhanced collaboration between food safety authorities, researchers, and the meat industry is essential to establish robust microbial monitoring systems and implement better hygienic practices to ensure consumer safety. The high counts of APC and PBC in this study highlight the importance of adhering to strict cold chain protocols and hygiene practices to minimize microbial growth. The detection of *E. coli* and coliforms further emphasizes the need for robust monitoring and interventions to prevent fecal contamination and cross-contamination, thereby safeguarding consumer health.

## 5. Conclusions

All examined local and imported meat samples exhibited unacceptable APC levels, exceeding the G.C.C. Standardization Organization (GSO) standards. This highlights the urgent need for improved hygiene practices in hypermarkets to reduce microbial contamination and ensure consumer safety. The similar bacterial contamination levels (APC, PBC, coliform, and *E. coli*) between local and imported samples suggest comparable hygienic conditions during handling and storage. However, significant variation in *E. coli* counts among hypermarkets underscores its potential as a reliable marker for identifying hygiene inconsistencies across retail sites. The detection of psychrotrophic spoilage bacteria (*P. luteola*, *P. fluorescens*, and *S. putrefaciens*) and pathogenic bacteria (*A. baumannii complex*, *A. viridans*, *E. faecalis*, and *O. ureolytica*) presents serious public health implications. High bacterial loads and spoilage organisms not only compromise meat quality but also pose risks of foodborne illnesses. This emphasizes the urgent need for rigorous monitoring systems and enhanced hygiene protocols across Oman’s meat supply chain. To address these challenges, we recommend establishing a structured and standardized monitoring protocol to ensure consistency in hygiene practices across all hypermarkets. This protocol should include routine microbial testing, the enforcement of food safety regulations, and the training of retail staff in proper meat handling procedures. Such a system will enable the identification and correction of hygiene deficiencies in real time, thereby mitigating public health risks.

## Figures and Tables

**Table 1:** Types of biochemical tests in 47 wells of the GN card used in VITEK 2 compact machine.

Well	Test	Abbreviation	Well	Test	Abbreviation
2	Ala-Phe-Pro-arylamidase	APPA	33	Saccharose/sucrose	SAC
3	Adonitol	ADO	34	D-Tagatose	dTAG
4	L-Pyrrolydonyl-arylamidase	PyrA	35	D-Trehalose	dTRE
5	L-Arabitol	IARL	36	Citrate (sodium)	CIT
7	D-Cellobiose	dCEL	37	Malonate	MNT
9	Beta-galactosidase	BGAL	39	5-Keto-D-gluconate	5KG
10	H2S Production	H2S	40	L-lactate alkalinization	ILATk
11	Beta-N-acetyl-glucosaminidase	BNAG	41	Alpha-glucosidase	AGLU
12	Glutamyl Arylamidase pNA	AGLTp	42	Succinate alkalinization	SUCT
13	D-glucose	dGLU	43	Beta-N-acetyl-galactosaminidase	NAGA
14	gamma-glutamyl-transferase	GGT	44	Alpha-galactosidase	AGAL
15	Fermentation/glucose	OFF	45	Phosphatase	PHOS
17	Beta-glucosidase	BGLU	46	Glycine arylamidase	GlyA
18	D-Maltose	dMAL	47	Ornithine decarboxylase	ODC
19	D-Mannitol	dMAN	48	Lysine decarboxylase	LDC
20	D-Mannose	dMNE	53	L-Histidine assimilation	IHISa
21	Beta-xylosidase	BXYL	56	coumarate	CMT
22	Beta-alanine arylamidase pNA	BAIap	57	Beta-glucuronidase	BGUR
23	L-Proline arylamidase	ProA	58	O/129 resistance	O129R
26	Lipase	LIP	59	Glu-Gly-Arg-Arylamidase	GGAA
27	Palatinose	PLE	61	L-Malate assimilation	IMLTa
29	Tyrosine arylamidase	TyrA	62	Ellman	ELLM
31	Urease	URE	64	L-Lactate assimilation	ILATa
32	D-Sorbitol	dSOR			

**Table 2:** Types of biochemical tests in 43 wells of the GP card used in VITEK 2 compact machine.

Well	Test	Abbreviation	Well	Test	Abbreviation
2	D-amygdalin	AMY	32	Polymixin B resistance	POLYB
4	Phosphatidylinositol phospholipase C	PIPLC	37	D-galactose	dGAL
5	D-xylose	dXYL	38	D-ribose	dRIB
8	Arginine dihydrolase 1	ADH1	39	L-lactate alkalinization	ILATk
9	Beta-galactosidase	BGAL	42	Lactose	LAC
11	Alpha-glucosidase	AGLU	44	N-acetyl-D-glucosamine	NAG
13	Ala-phe-pro arylamidase	APPA	45	D-maltose	dMAL
14	Cyclodextrin	CDEX	46	Bacitracin resistance	BACI
15	L-aspartate arylamidase	AspA	47	Novobiocin resistance	NOVO
16	Beta galactopyranosidase	BGAR	50	Growth in 6.5% nacl	NC6.5
17	Alpha-mannosidase	AMAN	52	D-mannitol	dMAN
19	Phosphatase	PHOS	53	D-mannose	dMNE
20	Leucine arylamidase	LeuA	54	Methyl-B-D-glucopyranoside	MBdG
23	L-proline arylamidase	ProA	56	Pullulan	PUL
24	Beta glucuronidase	BGURr	57	D-raffinose	dRAF
25	Alpha-galactosidase	AGAL	58	O/129 resistance	O129R
26	L-pyrrolydonyl-arylamidase	PyrA	59	Salicin	SAL
27	Beta-glucuronidase	BGUR	60	Saccharose/sucrose	SAC
28	Alanine arylamidase	AlaA	62	D-trehalose	dTRE
29	Tyrosine arylamidase	TyrA	63	Arginine dihydrolase 2	ADH2s
30	D-sorbitol	dSOR	64	Optochin resistance	OPTO
31	Urease	URE			

**Table 3:** Identification of bacteria isolated from fresh raw meat by VITEK.

Sample	Name	Bionumber	% Identification
Gram-negative			
13 (L)	*Acinetobacter baumannii complex* (*A. nosocomialis*, *A. pittii*, *A. baumannii*, *A. calcoaceticus*)	0203211301500210	93% (very good)
7 (I)	*Escherichia coli*	0405610540526611	99% (excellent)
8 (L)	*Escherichia coli*	0405610450026611	99% (excellent)
ATCC	*Escherichia coli* ATCC 25922	0405611560566601	98% (excellent)
Pistachio (I)	*Escherichia coli*	0405610450026611	99% (excellent)
12 (L)	*Oligella ureolytica*	0000001200000000	97% (excellent)
14 (I)	*Pseudomonas fluorescens*	4203251101500210	88% (acceptable)
13 (L)	*Pseudomonas fluorescens*	4203211101100210	89% (good)
2 (I)	*Pseudomonas luteola*	4207211301500250	85% (acceptable)
1 (I)	*Shewanella putrefaciens*	5030001100440000	96% (excellent)
2 (I)	*Shewanella putrefaciens*	5070001110440000	97% (excellent)
13 (L)	*Shewanella putrefaciens*	5050001100140001	98% (excellent)
Gram-positive			
12 (L)	*Aerococcus viridans*	000000200363430	87% (acceptable)
14 (I)	*Enterococcus faecalis*	176012665773671	94% (very good)
ATCC	*Staphylococcus aureus* ATCC 25923	030402067763231	99% (excellent)

L: local sample, I: imported sample. *E. coli* ATCC 25922 and *E. coli* isolated from pistachio were used for comparison and building a dendrogram for Gram-negative bacteria.

## Data Availability

The original contributions presented in this study are included in the article/Supplementary Materials, and further inquiries can be directed to the corresponding author.

## Supplementary Materials

The following supporting information can be downloaded at https://www.mdpi.com/article/10.3390/microorganisms12122545/s1, Figure S1: Steps to transform VITEK results of biochemical tests from a PDF file to an Excel file for further analysis.
