# Supplementary material for: Microbiological Evaluation of Local and Imported Raw Beef Meat at Retail Sites in Oman with Emphasis on Spoilage and Pathogenic Psychrotrophic Bacteria
Source: Microorganisms. 2024 Dec 11;12(12):2545. doi: 10.3390/microorganisms12122545 (PMC11679729; doi:10.3390/microorganisms12122545)
Supplement: Supplementary file 1 [file microorganisms-12-02545-s001.zip › microorganisms-3358688-supplementary.pdf]

In Excel click on:

1. Data
2. Get data
3. From file
4. From PDF

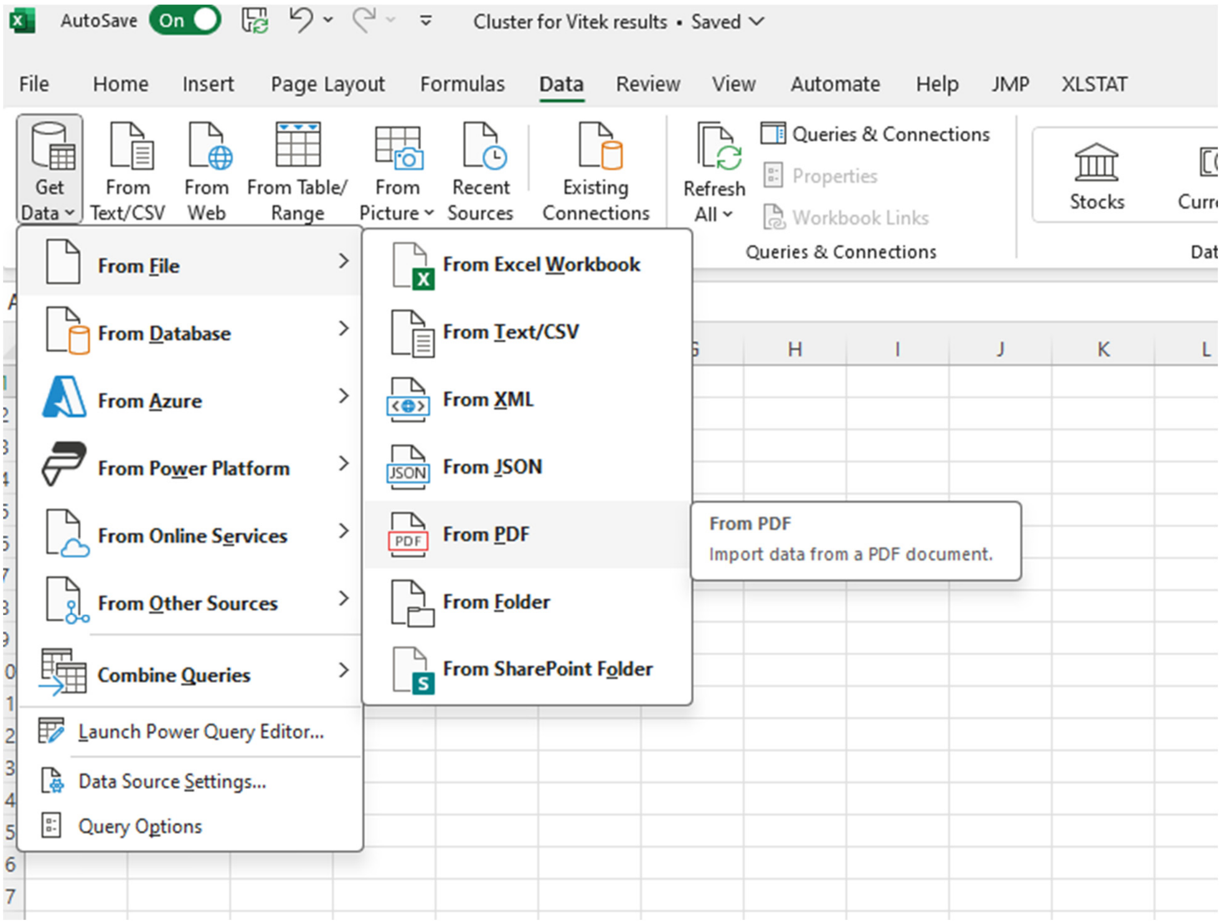

5. Choose correct PDF file
6. Import
7. Navigate to the correct table
8. Load

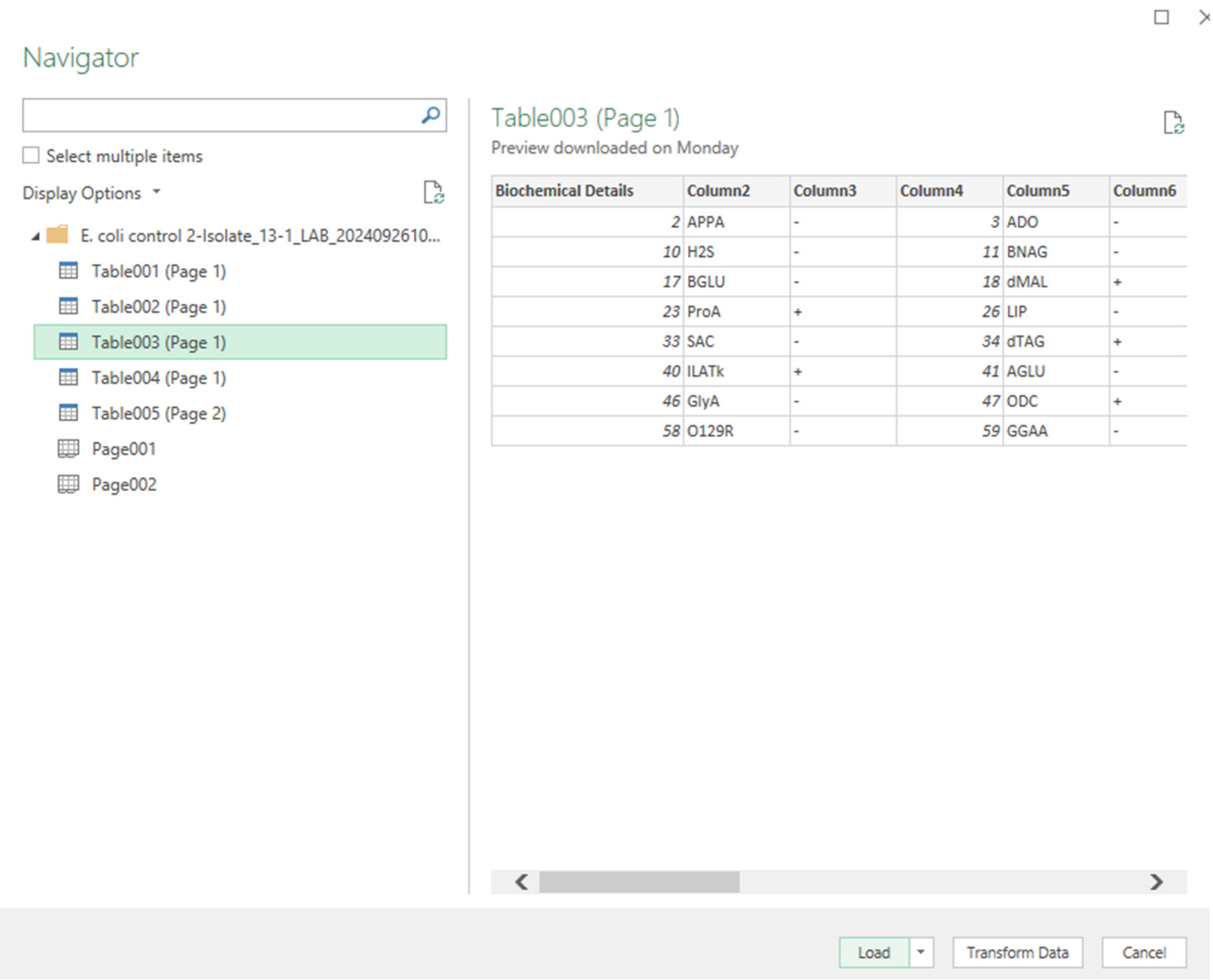

Biochemical Details	Column2	Column3	Column4	Column5	Column6
2	APPA	-	3	ADO	-
10	H2S	-	11	BNAG	-
17	BGLU	-	18	dMAL	+
23	ProA	+	26	LIP	-
33	SAC	-	34	dTAG	+
40	ILATk	+	41	AGLU	-
46	GlyA	-	47	ODC	+
58	O129R	-	59	GGAA	-

You will get something similar to this:

Biochemical Details	Column2	Column3	Column4	Column5	Column6	Column7	Column8	Column9	Column10	Column11	Column12	Column13	Column14	Column15	Column16	Column17	Column18
2	APPA	-	3	ADO	-	4	PyrA	-	5	lARL	-	7	dCEL	-	9	BGAL	+
10	H2S	-	11	BNAG	-	12	AGLTp	-	13	dGLU	+	14	GGT	-	15	OFF	+
17	BGLU	-	18	dMAL	+	19	dMAN	+	20	dMNE	+	21	BXYL	-	22	BAlap	-
23	ProA	+	26	LIP	-	27	PLE	-	29	TyrA	+	31	URE	-	32	dSOR	+
33	SAC	-	34	dTAG	+	35	dTRE	+	36	CIT	-	37	MNT	-	39	5KG	-
40	lLATk	+	41	AGLU	-	42	SUCT	+	43	NAGA	-	44	AGAL	+	45	PHOS	+
46	GlyA	-	47	ODC	+	48	LDC	+	53	lHISa	-	56	CMT	+	57	BGUR	+
58	O129R	-	59	GGAA	-	61	lMLTa	-	62	ELLM	+	64	lLATa	-			

9. Arrange all data in 2 columns (One for the tests names and one for the results (+ and -) using copy and paste functions)

Colum	Colum
APPA	-
H2S	-
BGLU	-
ProA	-
SAC	+
ILATk	-
GlyA	-
O129R	+
ADO	-
BNAG	-
dMAL	+
LIP	-
dTAG	-
AGLU	-
ODC	+
GGAA	-
PyrA	-
AGLTp	-
dMAN	+
PLE	-
dTRE	+
SUCT	-
LDC	+
lMLTa	-
lARL	-
dGLU	+
dMNE	+
TyrA	-
CIT	-
NAGA	-
lHISa	-
ELLM	+
dCEL	-
GGT	-
BXYL	-
URE	-
MNT	-
AGAL	+
CMT	+
lLATa	-
BGAL	+
OFF	+
BAlap	-
dSOR	+
5KG	-
PHOS	-
BGUR	+

10. Select the 2 columns
11. Copy
12. Paste special
13. Select transpose (the data should be organized horizontally). You can write the name of the corresponding bacteria at this stage.

Bacteria	APPA	H2S	BGLU	ProA	SAC	ILATk	GlyA	O129R	ADO	BNAG	dMAL	LIP	dTAG	AGLU	ODC	GGAA	PyrA	AGLTp	dMAN	PLE
Shewanella putrefaciens (S 1)	+	+	-	+	-	-	-	-	-	+	-	-	-	-	-	-	+	-	-	-

14. Repeat the above steps until you get the data for all bacteria.

A	B	C	D	E	F	G	H	I	J	K	L	M	N	O	P	Q
Bacteria	APPA	H2S	BGLU	ProA	SAC	ILATk	GlyA	O129R	ADO	BNAG	dMAL	LIP	dTAG	AGLU	ODC	GGAA
Shewanella putrefaciens (S 1)	+	+	-	+	-	-	-	-	-	+	-	-	-	-	-	-
Shewanella putrefaciens (S 2)	+	+	-	+	+	-	-	-	-	+	-	-	-	-	-	-
Pseudomonas luteola	-	-	-	+	-	+	-	+	-	-	+	-	-	-	-	-
Escherichia coli (S7)	-	-	-	-	-	+	-	+	-	-	+	-	-	-	+	-
Escherichia coli (S8)	-	-	-	-	+	-	-	+	-	-	+	-	-	-	+	-
Escherichia coli (ATCC 25922)	-	-	-	+	-	+	-	-	-	-	+	-	+	-	+	-
Escherichia coli (pistachio)	-	-	-	-	+	-	-	+	-	-	+	-	-	-	+	-

15. Select “replace” from the “find and select” function in Excel.
16. Write “-“ in “find what” and “0” in “replace with”.
17. Replace all. All negative results should change with 0 now.
18. Repeat the previous step to change all positive results (+) to (1).

Bacteria	APPA	H2S	BGLU	ProA	SAC	lLATk	GlyA	O129R	ADO	BNAG	dMAL	LIP	dTAG
Shewanella putrefaciens (S1)	1	1	0	1	0	0	0	0	0	1	0	0	0
Shewanella putrefaciens (S2)	1	1	0	1	1	0	0	0	0	1	0	0	0
Pseudomonas luteola (S2)	0	0	0	1	0	1	0	1	0	0	1	0	0
Escherichia coli (S7)	0	0	0	0	0	1	0	1	0	0	1	0	0
Escherichia coli (S8)	0	0	0	0	1	0	0	1	0	0	1	0	0
Escherichia coli (ATCC 25922)	0	0	0	1	0	1	0	0	0	0	1	0	1
Escherichia coli (pistachio)	0	0	0	0	1	0	0	1	0	0	1	0	0

19. The data are now ready to be analyzed by any statistical program for cluster relationships or any other analysis. This can include hundreds or thousands of data results.

Figure S1. Steps to transform VITEK results of biochemical tests from a PDF file to an Excel file for further analysis.
